# Tedizolid-Rifampicin Combination Prevents Rifampicin-Resistance on *in vitro* Model of *Staphylococcus aureus* Mature Biofilm

**DOI:** 10.3389/fmicb.2020.02085

**Published:** 2020-08-28

**Authors:** Anna Gidari, Samuele Sabbatini, Elisabetta Schiaroli, Stefano Perito, Daniela Francisci, Franco Baldelli, Claudia Monari

**Affiliations:** ^1^Department of Medicine, Clinic of Infectious Diseases, University of Perugia, Perugia, Italy; ^2^Department of Medicine, Medical Microbiology Section, University of Perugia, Perugia, Italy

**Keywords:** tedizolid, *S. aureus*, biofilm, rifampicin, daptomycin, antibiotic resistance

## Abstract

*Staphylococcus aureus* infections associated with implanted medical devices are difficult to treat and require long-lasting antibiotic therapies, especially when device removal is not possible or easy such as in the case of joint prostheses. Biofilm formation is a major cause of treatment failure and infection recurrence. This study aimed to test, for the first time, the *in vitro* combination of tedizolid plus rifampicin on methicillin-sensitive (MSSA ATCC 6538) and methicillin-resistant (MRSA ATCC 43300) *S. aureus* mature biofilm. Here, we demonstrated that the combination of tedizolid with rifampicin significantly disaggregated pre-formed biofilm of both strains, reduced their metabolic activity and exerted bactericidal activity at clinically meaningful concentrations. Notably, tedizolid was able to completely prevent the emergence of resistance to rifampicin. Moreover these effects were similar to those obtained with daptomycin plus rifampicin, a well-known and widely used combination. Preliminary results on some MRSA clinical isolates confirmed the efficacy of this combination in reducing biofilm biomass and preventing rifampicin resistance onset. Further *in vivo* studies are needed to confirm the validity of this promising therapeutic option that can be useful against biofilm-associated *S. aureus* infections.

## Introduction

*Staphylococcus aureus* is one of most frequently encountered bacterial species forming biofilms on medical devices, such as venous central or peripheral catheters, bladder catheters, and joint and valve prostheses. It causes difficult to treat infections especially when it is not possible, or not easy, to remove the device, such as in the case of joint or heart valves prostheses (Figueiredo et al., 2017). The etiology depends on several factors, including the timing of infections with respect to joint replacement (Coventry, 1975; Fitzgerald, 1989; Tsukayama et al., 1996; Zimmerli et al., 2004). *S. aureus* is the most frequent pathogen responsible for early-onset infections that occur <3 months after the surgery (Coventry, 1975). Delayed-onset infections that occur 3–12 months after surgery are typically caused by less virulent pathogens. Late-onset infections (>12 months after surgery) are generally the result of bloodstream dissemination. In all these cases, antibiotic therapy exerts a primary role and has to be administered for long periods, whether or not surgery is involved (Osmon et al., 2013). Therefore, an important issue of modern medicine is to guarantee effective home care both for reducing costs and improving patient compliance.

Rifampicin is one of the most effective antibiotics when used in combination with other anti-staphylococcal agents against staphylococcal biofilms *in vitro*. However, as a single agent, rifampicin does not effectively destroy biofilm due to the emergence of antibiotic resistance (Raad et al., 2007; Lima-e-Silva et al., 2017; Maudsdotter et al., 2019). The excellent efficacy of rifampicin against staphylococcal biofilm *in vitro* was confirmed by animal models and in patients with orthopedic device-related infections undergoing debridement and implant retention (Zimmerli and Sendi, 2019).

Daptomycin is a glycopeptide that is widely used in staphylococcal infections, including complicated systemic ones. It is active on MRSA strains and resistance onset is uncommon (Heidary et al., 2018). Daptomycin has shown modest activity *in vitro* against biofilm when used alone, but in combination with rifampicin it has been shown to significantly inhibit resistance onset to rifampicin (Boudjemaa et al., 2016).

Tedizolid phosphate is a new oxazolidinone pro-drug that is converted to the active drug tedizolid (TR-700) in serum. It acts by inhibiting protein synthesis and has a broad range of activity against Gram-positive pathogens, including linezolid-resistant strains (Kanafani and Corey, 2012). Moreover, tedizolid exhibited a four-fold greater potency compared to linezolid (Brown and Traczewski, 2010; Prokocimer et al., 2012). Tedizolid demonstrated *in vitro* and *in vivo* efficacy against MSSA and MRSA strains, showing strong antibiotic activity (Louie et al., 2011; Pfaller et al., 2019).

Our study aimed to test the activity of the above mentioned antibiotics in an *in vitro* model of mature *S. aureus* preformed biofilm. In particular, we tested the combination of tedizolid plus rifampicin and daptomycin plus rifampicin, a well-known and widely used association against infections caused by *S. aureus*. We evaluated the effect of these two combinations on biofilm biomass, viability, and, in particular, on rifampicin resistance onset. In addition, preliminary experiments on 3 MRSA clinical isolates were included in this study to confirm the efficacy of tedizolid/rifampicin combination in reducing biofilm biomass and, above all, in preventing the rifampicin resistance onset.

## Materials and Methods

### Microorganisms and Antimicrobial Agents

Methicillin-sensitive *S. aureus* (MSSA) ATCC 6538 and methicillin-resistant *S. aureus* (MRSA) ATCC 43300 were purchased from the American Type Culture Collection for use in this study. Three MRSA clinical strains isolated from patients with biofilm-related bloodstream infections were included in the study. Glycerol stocks were stored at −80°C. Before each experiment, bacteria were grown overnight at 37°C on trypticase soy agar (TSA) plates. For biofilm assays, bacterial suspensions were prepared in supplemented trypticase soy broth (sTSB) with 1% glucose (Sigma) and 0.5% NaCl (Sigma). Tedizolid was purchased from MSD Italia. Rifampicin and daptomycin powders were purchased from Sigma. Stock solutions were prepared according to the manufacturer’s instructions and small aliquots were stored at −80°C. Antibiotics were thawed immediately before each treatment.

### Susceptibility Testing

The minimal inhibitory concentrations (MICs) of the antimicrobial agents were determined by the broth microdilution method according to the European Committee on Antimicrobial Susceptibility Testing (EUCAST) guidelines (EUCAST, 2019). Antimicrobial susceptibility of the 3 MRSA clinical isolates were determined using the VITEK^®^ 2 System (BioMérieux, France) and interpreted according to EUCAST guidelines (EUCAST, 2019).

### *In vitro* Biofilm Formation and Quantification

Biofilm formation was carried out according to the method described by Bauer et al. (2013) with some modifications. Briefly, biofilms were grown in 96-well flat bottom plates (Corning) with an initial inoculum of 1 × 10^7^ CFU/mL (200 μL/well) in sTSB. Plates were incubated for up to 96 h at 30°C and, in parallel experiments, spent medium was renewed every 24 h. The total biofilm biomass was quantified after 24, 48, and 72 h of incubation using the crystal violet (CV) staining method. After the incubation period, each well of the plates was washed twice with 200 μL of phosphate-buffered saline (PBS, Gibco, United Kingdom) to remove non-adherent and loosely attached cells. Then, biofilms were fixed with 200 μL of 99% methanol (VWR Chemicals, France) for 15 min at room temperature (RT). After that, plates were left to dry before the staining with 200 μL of 0.1% CV solution for 20 min at RT. Finally, wells were washed with 200 μL of PBS to remove excess stain and the dye fixed to biofilms was solubilized with 200 μL of 33% glacial acetic acid (AppliChem, Germany). After 1 h of RT incubation without shaking, CV absorbance was measured at 590 nm with a microplate spectrophotometer (Tecan Infinite M200, Tecan Trading AG, Switzerland) (Stepanović et al., 2000). The 2,3-bis-(2-methoxy-4-nitro-5-sulfophenyl)-2H-tetrazolium-5-carboxanilidereduction (XTT) assay was used as previously described (Cerca et al., 2005). Fresh XTT (Invitrogen, United States) was prepared in PBS at a final concentration of 1 mg/mL and stored at −80°C. A phenazine methosulphate (PMS) solution was prepared at 1 mg/mL in sterile water. After the biofilms washings, 200 μL of an XTT-PMS solution (200 μg/mL XTT and 20 μg/mL PMS) was added to each well of the plate and incubated for 2 h at 37°C in the dark. Absorbance was measured at 450 nm using a reference filter at 690 nm.

### Antibiotic Activity on Mature *S. aureus* Biofilm

*Staphylococcus aureus* biofilms were grown as previously described (Bauer et al., 2013). Briefly, mature 48-h-old biofilms were incubated for further 48 h with sTSB containing serial dilutions of the three antimicrobial agents either alone or in combination. The media containing antibiotics were renewed after 24 h of incubation. Experiments with rifampicin alone were carried out until 72 h of incubation to evaluate the kinetics of resistance onset. After treatments, the biofilms were washed twice and the biomass was assessed as previously described (Stepanović et al., 2000). The metabolic activity of biofilms was determined by the XTT reduction assay as described above (Cerca et al., 2005), and biofilms were suspended in 100 μL of sterile PBS by scraping the wells with sterile tips and the suspensions were used for the colony-forming unit (CFU) count. Bacterial suspensions were vigorously vortexed and serial 10-fold dilutions were then performed. Then, three drops (10 μL) of suspension per dilution were deposited on TSA plates, and the plates incubated at 37°C for 24 h (Alves et al., 2018). The detection limit of countable bacteria was 2 log_10_ CFU/well. The bactericidal and bacteriostatic activities of the antibiotics alone and in combination were evaluated as previously described (Parra-Ruiz et al., 2010). Bactericidal and bacteriostatic effects were defined as a reduction in the CFU count of ≥3 log_10_ CFU/well and <3 log_10_ CFU/well compared to the control, respectively. The activity of antibiotic combinations was considered enhanced or improved if the reduction in the CFU count was ≥2 log_10_ CFU/well or 1 to 2 log_10_ CFU/well, respectively, compared to the most active single antimicrobial agent of the combination (Parra-Ruiz et al., 2010; Barber et al., 2015).

Biofilms that were not exposed to antimicrobial agents were used as control biofilms, and negative controls were wells containing medium.

### Rifampicin-Resistant Mutants

To determine the onset of rifampicin resistance, biofilms were plated both on TSA plates with and without 20 mg/L rifampicin (Boudjemaa et al., 2016). The percentage of rifampicin-resistant mutants was calculated using the ratio of CFU grown with and without the antibiotic. After incubations, colonies growing on antibiotic-supplemented medium were subjected to antimicrobial susceptibility test and rifampicin resistance was confirmed by MIC determination according to EUCAST guidelines (EUCAST, 2019).

### Statistical Analysis

All analyses were performed using Prism Graphpad 7 software. Data are summarized as the means ± SEM of three independent experiments performed in triplicate. Data with normal distribution were analyzed with one-way analysis of variance (ANOVA) and Bonferroni’s multiple-comparison test. For nonparametric variables, the Kruskal–Wallis test and Dunn’s multiple comparison test were performed. A *p*-value <0.05 was considered statistically significant.

## Results

### Susceptibility Testing

The strains were found to be equally susceptible to tedizolid and daptomycin and both these drugs had the same MIC of 0.25 mg/L. The MIC of rifampicin was 0.002 mg/L and 0.001 mg/L for MSSA and for MRSA, respectively. ATCC reference strains and MRSA clinical isolates were all susceptible to tedizolid, rifampicin and daptomycin (EUCAST, 2019).

### *In vitro* Biofilm Formation and Quantification

**FIGURE 1:**
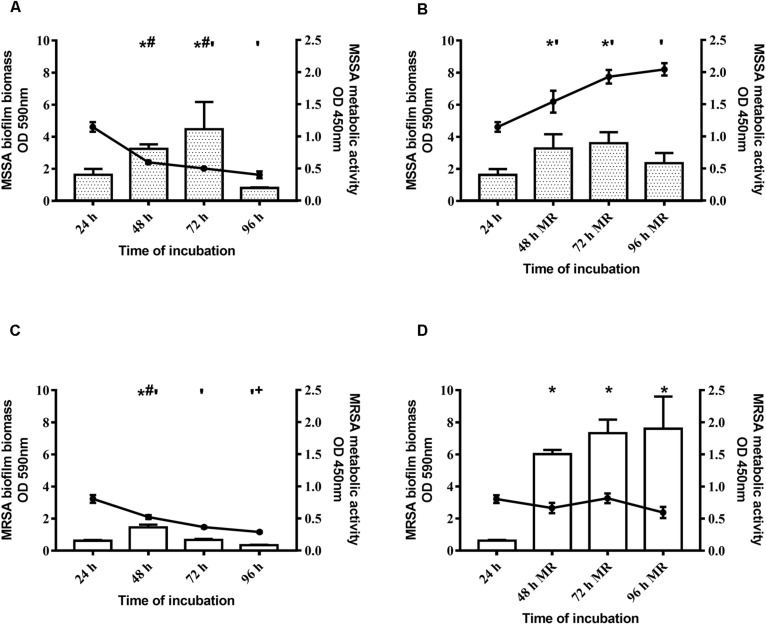
Time course of biofilm biomass and metabolic activity of two *Staphylococcus aureus* strains. The biofilm biomass was evaluated using the crystal violet staining method (OD 590 nm, bars) and metabolic activity was determined using the XTT reduction assay (OD 450 nm, lines with filled circles). **(A,C)** Methicillin-sensitive *Staphylococcus aureus* (MSSA) ATCC 6538 and methicillin-resistant *S. aureus* (MRSA) ATCC 43300 were incubated for 96 h and the biofilm biomass and metabolic activity were assessed at selected time points without changing the spent medium or **(B,D)** renewing the medium every 24 h (MR = Medium Renewal). Data are expressed as the means ± SEM of three independent experiments in triplicate. Statistically significant differences were tested with ordinary one-way ANOVA or Kruskal–Wallis test. *p < 0.05 biofilm biomass vs biofilm biomass at 24 h. ^#^*p* < 0.05 biofilm biomass vs biofilm biomass at 96 h, ‘*p* < 0.05 biofilm metabolic activity vs biofilm metabolic activity at 24 h. ^+^*p* < 0.05 biofilm metabolic activity vs biofilm metabolic activity at 48 h.

With regard to the kinetics of biofilm formation of MSSA and MRSA, without changing the medium the biomass of both strains peaked at 48–72 h, and then decreased (Figures 1A,C). By renewing the medium every 24 h, the biomass increased until 48 h and then remained stable until 96 h (Figures 1B,D). Figure 1 (panels A and C) shows, also, that metabolic activity of both strains peaked at 24 h, and progressively reduced thereafter. By renewing the medium, the metabolic activity of both strains increased up to 48 h and then remained steady until 96 h (Figures 1B,D). The biofilm obtained after 48 h of incubation by renewing the medium after 24 h was considered mature biofilm and this was used for the subsequent evaluations.

### Effect of Rifampicin Treatment on Mature MSSA and MRSA Biofilms

**FIGURE 2:**
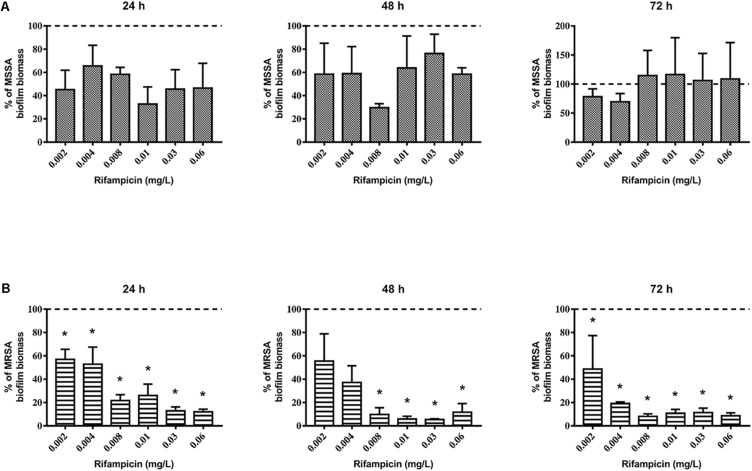
Biofilm biomass of 48-h-old biofilm of *Staphylococcus aureus* **(A)** ATCC 6538 and **(B)** ATCC 43300 after rifampicin treatment. Biofilm biomass was evaluated using the crystal violet staining method after 24, 48, and 72 h of treatment with different concentrations of rifampicin. Results from three independent experiments in triplicate are expressed as percentage ± SEM of biofilm biomass with respect to the untreated control biofilm (dashed line). Statistically significant differences were tested with ordinary one-way ANOVA or Kruskal–Wallis test. *p < 0.05 rifampicin-treated biofilm vs untreated control biofilm.

**FIGURE 3:**
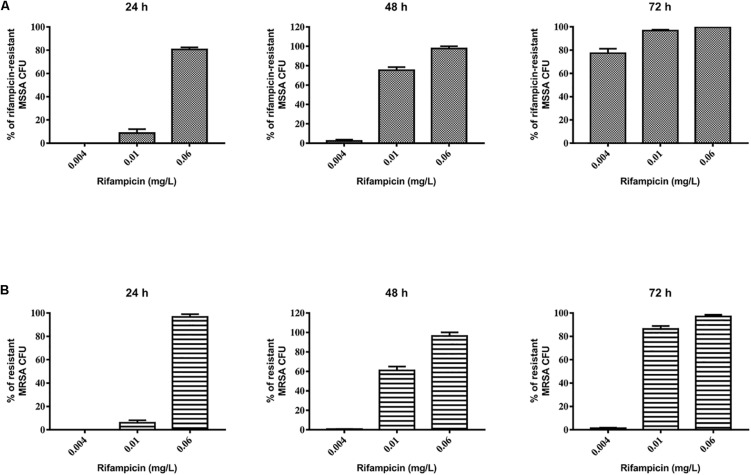
Evaluation of rifampicin-resistant CFU of 48-h-old biofilm of *Staphylococcus aureus* **(A)** ATCC 6538 and **(B)** ATCC 43300 after treatment with rifampicin. Rifampicin-resistant CFU were evaluated by plating biofilm onto TSA plates containing 20 μg/mL of rifampicin after treatment for 24, 48, and 72 h with selected concentrations of rifampicin. The results from three independent experiments in triplicate are expressed as percentages ± SEM of rifampicin-resistant CFU after treatment.

First, we evaluated the activity of rifampicin alone on mature biofilm. To this end, MSSA and MRSA biofilms were treated for 24, 48, and 72 h with 0.002–0.06 mg/L of rifampicin. Our results show that there were not significant differences in the biomass of the untreated control compared to that of rifampicin-treated MSSA biofilm (Figure 2A), whereas rifampicin reduced the biofilm biomass of MRSA especially at antibiotic concentrations ranging from 0.008 to 0.06 mg/L after 48 and 72 h of treatment (Figure 2B). Rifampicin-resistant strains were found from 24 h onward both on MSSA and MRSA biofilms, and the onset of resistance was dose-dependent. In fact, higher concentrations of rifampicin were able to induce rifampicin-resistant mutants at a faster rate than lower concentrations of rifampicin (Figures 3A,B).

Based on these results, we selected 0.06 mg/L rifampicin for subsequent experiments as, at this concentration, almost 100% of rifampicin-resistant strains were obtained in both staphylococcal biofilms after 48 h of treatment. Rifampicin resistance was confirmed by antimicrobial susceptibility assay: all tested colonies were rifampicin resistant (MIC >0.5 mg/L).

### Effect of Tedizolid-Rifampicin Treatment on Mature MSSA and MRSA Biofilms

Next, we evaluated the effect of the combination of tedizolid-rifampicin on 48-h-old MSSA and MRSA biofilms. The combination daptomycin-rifampicin has been included as a positive control according to previous data supporting the efficacy in biofilm-related infections (LaPlante and Woodmansee, 2009; Hall Snyder et al., 2015; Telles et al., 2019; Kamble and Pardesi, 2020).

**FIGURE 4:**
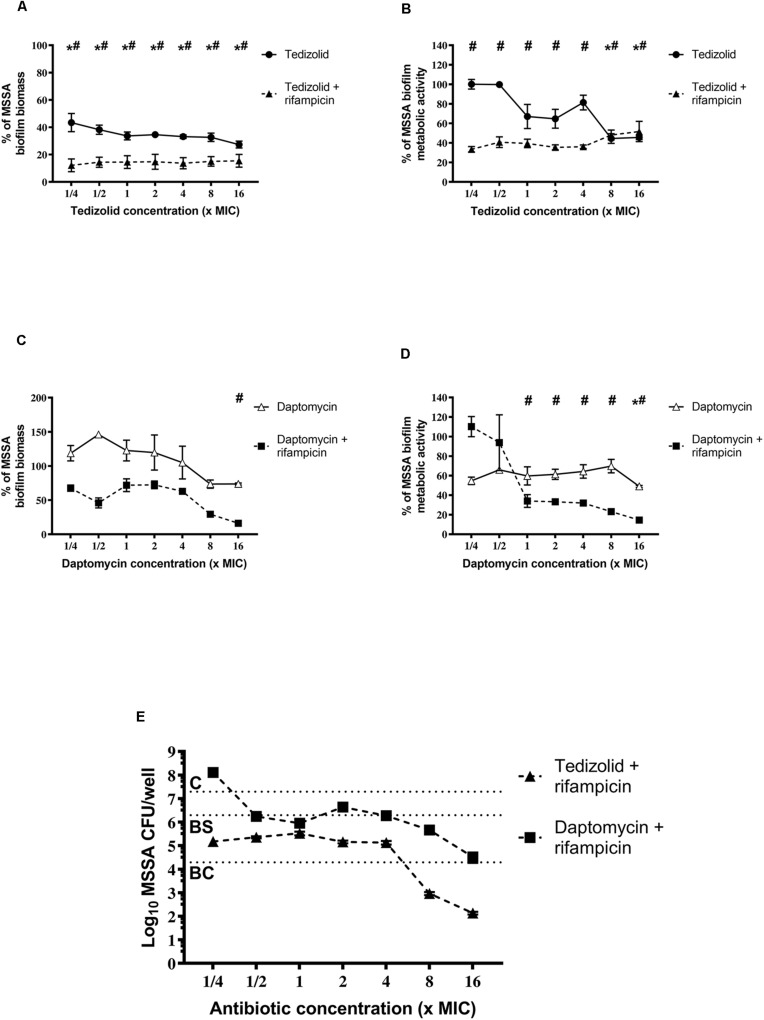
Biofilm biomass, metabolic activity, and CFU analysis of 48-h-old *Staphylococcus aureus* ATCC 6538 biofilm after treatment with different concentrations of single/combined antibiotics. The biofilm biomass and metabolic activity were evaluated using crystal violet staining and the XTT reduction assay, respectively, after 48 h of treatment with **(A,B)** tedizolid alone (lines with filled dots) or in combination with rifampicin (dashed lines with filled triangles) and **(C,D)** daptomycin alone (lines with empty triangles) or in combination with rifampicin (dashed lines with filled squares). Results from three independent experiments in triplicate are expressed as percentage ± SEM with respect to the untreated control biofilm. Statistically significant differences were tested with ordinary one-way ANOVA or Kruskal–Wallis test. *p < 0.05 biofilm treated with single antibiotic vs untreated control biofilm. ^#^*p* < 0.05 biofilm treated with combination of antibiotics vs untreated control biofilm. **(E)** Evaluation of bacteriostatic and bactericidal activities of biofilms treated with various antibiotic combinations (C = control untreated biofilm, BS = bacteriostatic effect threshold, BC = bactericidal effect threshold).

Our results show that tedizolid (1/4–16× the MIC) used in combination with rifampicin induced a significant reduction of MSSA biofilm biomass (Figure 4A) as well as of metabolic activity at all the concentrations tested (Figure 4B), compared to untreated biofilm. Tedizolid alone reduced the biomass with a trend similar to that observed in combination, whereas it reduced the metabolic activity only at the two highest concentrations used (8–16× the MIC; Figures 4A,B). The combination of daptomycin (1/4–16× the MIC) with rifampicin showed a significant reduction of biofilm biomass only at the dose of 16× the MIC (Figure 4C) and of metabolic activity at the doses of 1-16× the MIC (Figure 4D). Daptomycin alone did not produced considerable effects (Figures 4C,D). Data obtained from the CFU count (Figure 4E) show that: the combination tedizolid plus rifampicin, with 8–16× the MIC of tedizolid (average [AVG] –Δ4.33 and 5.16 log_10_ CFU/well, respectively) was able to achieve bactericidal effect and with 2–4× the MIC of tedizolid produced bacteriostatic effect (AVG –Δ2.13 and 2.16 log_10_ CFU/well); rifampicin plus daptomycin exhibited a bacteriostatic effect when daptomycin concentrations were 8–16× the MIC (AVG –Δ1.63 and 2.77 log_10_ CFU/well, respectively). Further, compared to rifampicin alone, the association with tedizolid (8–16× the MIC) showed enhanced activity (AVG –Δ2.14 and 3.97 log_10_ CFU/well, respectively) while the association with daptomycin (16× the MIC) improved the activity (AVG –Δ1.6 log_10_ CFU/well) of single antibiotics.

**FIGURE 5:**
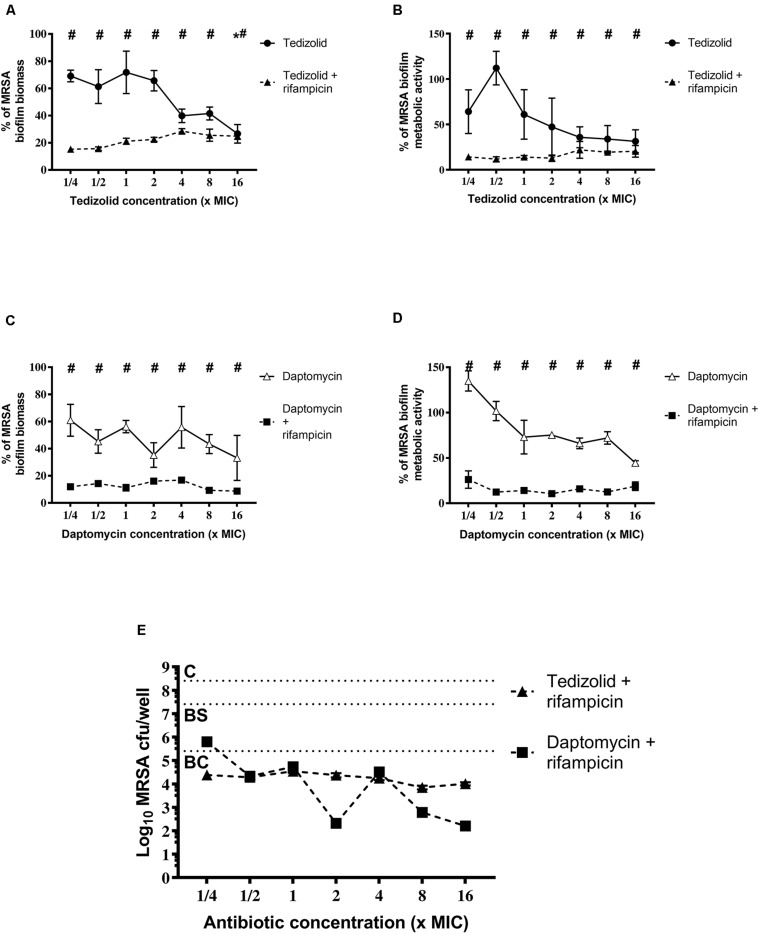
Biofilm biomass, metabolic activity, and CFU analysis of 48-h-old biofilm of *Staphylococcus aureus* ATCC 43300 after treatment with different concentrations of antibiotics used either alone or in combination. The biofilm biomass and metabolic activity were evaluated using the crystal violet staining method and the XTT reduction assay, respectively, after 48 h of treatment with **(A,B)** tedizolid alone (lines with filled dots) or in combination with rifampicin (dashed lines with filled triangles) and **(C,D)** daptomycin alone (lines with empty triangles) or in combination with rifampicin (dashed lines with filled squares). Results from three independent experiments in triplicate are expressed as percentages ± SEM with respect to the untreated control biofilm. Statistically significant differences were tested with ordinary one-way ANOVA or Kruskal–Wallis test. *p < 0.05 biofilm treated with single antibiotic vs untreated control biofilm. ^#^*p* < 0.05 biofilm treated with a combination of antibiotics vs untreated control biofilm. **(E)** Evaluation of bacteriostatic and bactericidal activity of the biofilms treated with various antibiotic combinations (C = control untreated biofilm, BS = bacteriostatic effect threshold, BC = bactericidal effect threshold).

Concerning the effect on MRSA biofilm tedizolid plus rifampicin resulted in a significant reduction in the biomass as well as metabolic activity (Figures 5A-D). Similar trend was observed in the presence of daptomycin plus rifampicin. Tedizolid alone significantly reduced the MRSA biomass at the highest concentration tested (16× the MIC; Figure 5A) whereas daptomycin alone led to no differences in biomass compared to the control (Figure 5C). The CFU count (Figure 5E) showed a bactericidal effect for both the combinations: tedizolid plus rifampicin at all the concentrations tested (AVG –Δ4.02 to 4.55 log_10_ CFU/well), daptomycin plus rifampicin when daptomycin was used at 1/2–16× the MIC (AVG –Δ4.08 to 6.20 log_10_ CFU/well). Further, compared to rifampicin alone, the combination with tedizolid and daptomycin showed enhanced activity at all the concentrations of tedizolid used (AVG –Δ2.07 to 2.45 log_10_ CFU/well) and at 2–16× the MIC of daptomycin (AVG –Δ2.13 to 4.25 log_10_ CFU/well).

### Impact of Tedizolid-Rifampicin and Daptomycin-Rifampicin Combination on Rifampicin Resistance Emergence

**FIGURE 6:**
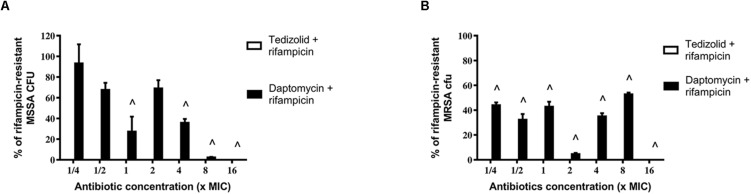
Percentage of rifampicin-resistant CFU of 48-h-old biofilm of *Staphylococcus aureus* **(A)** ATCC 6538 and **(B)** ATCC 43300 after treatment with different concentrations of antibiotics in combination. Rifampicin-resistant CFU were evaluated by plating biofilm onto TSA plates containing 20 μg/mL rifampicin after 48 h of treatment with the rifampicin-daptomycin and rifampicin-tedizolid combinations. Results from three independent experiments in triplicate are expressed as percentages ± SEM of rifampicin-resistant CFU after treatment. Statistically significant differences were tested with ordinary one-way ANOVA or Kruskal–Wallis test. ^∧^*p* < 0.05 antibiotic combination-treated biofilm rifampicin-resistant CFU vs rifampicin-treated biofilm rifampicin-resistant CFU. Values for all tedizolid + rifampicin combinations are zero, so the bars are not visible in the graph.

After treatments, biofilms were analyzed for rifampicin resistance onset. Regarding the MSSA strain, the combination of tedizolid and rifampicin completely inhibited the onset of rifampicin resistance at all the concentrations used, whereas daptomycin and rifampicin significantly reduced the resistance onset when daptomycin was used at ≥1× the MIC (Figure 6A).

Of note, also in the case of MRSA strain tedizolid plus rifampicin completely abolished the onset of rifampicin resistance for all doses tested and daptomycin plus rifampicin significantly decreased the onset of rifampicin resistance for every concentration tested (Figure 6B). Daptomycin (16× the MIC) combined with rifampicin was able to completely inhibit resistance both in the MSSA that in the MRSA strain (Figures 6A,B).

### Effect of Tedizolid-Rifampicin Treatment on MRSA Clinical Isolates

Biofilm of three MRSA clinical isolates was treated following the same experimental design as for reference strains. Two tedizolid concentrations (2 and 4 mg/L), corresponding to the two higher concentrations used for reference strains, were selected according to clinically meaningful concentrations reachable *in vivo*, and combined with rifampicin as above described. Results of representative experiment are reported in Table 1. The mean percentages of biofilm biomass of clinical isolates treated with tedizolid (2 and 4 mg/L) plus rifampicin (0.06 mg/L) were about 65 and 55%, respectively, compared to that of the untreated biofilm. Rifampicin treatment led to resistance onset in all selected strains, whereas, of note, the combination of tedizolid and rifampicin was able to prevent the rifampicin resistance onset in all the strains tested, mirroring the effects previously obtained with reference strains.

**TABLE 1:** Effect of tedizolid/rifampicin combination on MRSA clinical isolates.

	Rifampicin + tedizolid (2 mg/L)		Rifampicin + tedizolid (4 mg/L)	
		
	% of biofilm biomass	% of rifampicin-resistant strains	% of biofilm biomass	% of rifampicin-resistant strains
**MRSA Isolate 1**	67.64.1	0	61.52.8	0
**MRSA Isolate 2**	58.93.8	0	40.74.7	0
**MRSA Isolate 3**	73.13.8	0	62.52.7	0

*Percentages of biofilm biomass and rifampicin-resistant CFU of 48-h-old biofilm of 3 clinically isolated MRSA strains after 48 h of treatment with two combinations of tedizolid plus rifampicin. Biofilm biomass was evaluated using the crystal violet staining method and compared to untreated controls. Rifampicin-resistant CFU were evaluated by plating biofilm onto TSA plates containing 20 μg/mL rifampicin. Results are expressed as mean ± SEM of a single experiment with three technical replicates. Abbreviations: MRSA, methicillin-resistant *Staphylococcus aureus*.*

## Discussion

Nowadays, there is widespread use of different kinds of devices in clinical practice. These devices are doubtless necessary, but are often associated with difficult-to-treat infections (Mermel, 2017). Meta-analytical studies estimate that bloodstream infections (BSIs) are the fifth leading cause of hospital-acquired infections, with mortality rates of 12–25% and *S. aureus* is the leading pathogen in about 10% of the cases (Maki et al., 2006; Monegro and Regunath, 2020). *S. aureus* is responsible of several life-threatening infections and increase in the annual mortality rate if the infection is associated with medical devices due to *S. aureus* biofilm, which reduces antibiotic penetration. Another important issue is that infections related to medical devices such as prosthetic joints, which need suppression therapy, are difficult to treat. In this setting, oral antibiotic therapy becomes the primary mode of treatment and is indispensable.

Rifampicin in combination with other anti-staphylococcal agents, such as daptomycin, is highly recommended in infections involving prosthetic devices (Osmon et al., 2013; Baddour et al., 2015; Boudjemaa et al., 2016). This kind of infections requires long-term antibiotic treatments and, as in the case of daptomycin, intravenous administered therapies must be guaranteed by healthcare professionals. Tedizolid is a new oxazolidinone that has recently been approved to treat acute skin and soft tissue infections. To date there are limited and conflicting data about its activity on biofilms (Bayer et al., 2016; Delpech et al., 2018; Abad et al., 2019), and there are no data available about its activity in combination with rifampicin on *S. aureus* biofilm. Indeed, to our knowledge, only Werth (2017) evaluated the *in vitro* pharmacodynamic interactions between tedizolid and rifampicin, and Park et al., 2016 evaluated the effect of this combination on an *in vivo* model of methicillin-resistant *S. aureus* foreign body-associated osteomyelitis. The first study focused the effect on *S. aureus* planktonic cells and among the combinations tested tedizolid/rifampicin seemed the most likely one to improve activity, but synergy was not found in every strain. Otherwise, in the *in vivo* model the combinatory treatments tedizolid plus rifampicin and vancomycin plus rifampicin were both effective in reducing bone tissue infection and were not statistically significant differences between them. To notice that all the groups were found to have rifampicin-resistant mutants after treatments. Regarding tedizolid plus rifampicin treatment group, the authors stated that the low trough level of rifampicin found after 3 weeks of treatment may have contributed to the phenomenon (Park et al., 2016).

Our study is the first one to evaluate the activity of the association tedizolid plus rifampicin against *S. aureus* mature biofilm *in vitro*. We decided to treat a mature and stable biofilm, which did not decrease or increase both as biomass and metabolic activity. The medium was renewed every 24 h in both the biofilm formation and drug treatment experiments for the following reasons: to ensure that the biofilm was in the most favorable conditions of growth and nutrients were always available and to avoid an eventual disaggregation or decline of metabolic activity due to unfavorable conditions rather than due to antibiotic activity. Furthermore, although the limits of a static *in vitro* system must be accounted for, this system is more similar to *in vivo* conditions. In this study, we used two different sets of *S. aureus* strains, MSSA and MRSA, based on the well-known differences that the *mecA* gene bestows to the pathogen. In fact, studies on the fitness of MRSA strains have revealed a reduction in the growth rates of these strains as a cost to pay to survive under antibiotic pressure (Ender et al., 2004; Nielsen et al., 2012). Furthermore, it was not certain that both types of strains would have had the same response to antibiotic therapy and the biofilm formation has been shown to be substantially different in MSSA and MRSA strains (McCarthy et al., 2015). Here, on our *in vitro* experimental model, we demonstrated that the combination of rifampicin and tedizolid has a significant activity against both MSSA and MRSA pre-formed biofilms, despite the strains showed different fitness in terms of biofilm biomass and metabolic activity. Of note, this effect is associated with tedizolid capacity to prevent rifampicin resistance onset and it is similar to that of rifampicin and daptomycin. These results are discordant with Park et al. (2016) ones, but the models are not fully comparable. Indeed, we used a static *in vitro* model in which antibiotic concentrations are maintained stable while Park’s model is a dynamic *in vivo* one that takes in account pharmacokinetics fluctuations. Tedizolid plus rifampicin is able to significantly disaggregate biofilm-embedded bacteria even at tedizolid sub-optimal concentrations and it shows bactericidal activity at clinically meaningful concentrations. Especially MRSA ATCC strain seems to be more affected by the combinatory treatment. This result is particularly important since the acquisition of *rpoB* gene mutations, that are responsible of rifampicin resistance (Donlan and Costerton, 2002; Villar et al., 2011; Ryder et al., 2012; Zhou et al., 2012), has been found to confer reduced susceptibility to other drugs giving rise to serious treats in clinical settings (Bongiorno et al., 2018).

Since it is well known that clinically obtained strains usually show different behaviors following antibiotic treatments, three clinical MRSA isolates from patients with biofilm-related bloodstream infections were included in this study in order to obtain preliminary results about the effect of rifampicin-tedizolid combination. As expected, rifampicin treatment alone was able to induce resistance in all the strains. Taken together, the results showed that clinical isolates are affected by tedizolid (at the human serum peak range concentrations) combined with the selected rifampicin dose. Even if the obtained effect (nearly 50% of biomass reduction) results quite different to that observed against reference MRSA strain, it should be taken into account that the concentration of rifampicin used in our study was selected in order to study the effect of the combination on rifampicin resistance inhibition. Higher rifampicin concentration should be used to have a more accurate overview of the real anti-biofilm efficacy of this combination. Regardless of its effectiveness, the most important result achieved was the ability of tedizolid to totally avoid the emergence of rifampicin-resistant strains following the combinatory treatment.

Our results on *S. aureus* biofilm, together with previously available data (Bassetti et al., 2019; Carvalhaes et al., 2019), suggest a complementary activity of this combination. Indeed, rifampicin is known to be able to disaggregate *S. aureus* biofilm, while tedizolid has a good antimicrobial activity against planktonic bacteria and it has been demonstrated that inhibits *S. aureus* biofilm formation (Delpech et al., 2018; Abad et al., 2019). So, it is easy to speculate that rifampicin and tedizolid combination has biofilm-disrupting ability and that could also prevent dissemination and subsequent biofilm regrowth from biofilm-detached cells; this is a usually encountered complication in biofilm-related infections leading to treatment failure and infections recurrences.

The limitations of our study are the use of only two reference strains and three clinical isolates due to the wide variability of response of the single strain, although the same strain might have similar *in vitro* susceptibility and a static *in vitro* model was used, which does not account for most of the pharmacokinetic aspects. For example, the *in vivo* concentrations of daptomycin reported elsewhere were considerably higher than those used in our study (Dvorchik et al., 2003). On the other hand, the tedizolid concentrations used in this study correspond to the concentrations reached *in vivo*.

In conclusion, this study demonstrated the effectiveness of an antibiotic combination that could be used against difficult-to-treat infections. Indeed, tedizolid and rifampicin are antibiotics that can be administered via both the intravenous and oral routes. Therefore, their association could be interesting especially for patients who need long-term antibiotic therapy. Even if the prolonged exposure to rifampicin could bring to slight resistance onset, we may suppose that tedizolid could maintain its efficacy on biofilm disaggregation and regrowth inhibition after mutation. This could be somehow ascribed to the similar mechanism of action of both antimicrobials that inhibit protein synthesis (Falagas et al., 2007; Abad et al., 2019). Based on our results and hypotheses, further experiments will be established to deeply study the effect of tedizolid and rifampicin combination on clinically isolated strains. Additionally, rifampicin-resistant *S. aureus* strains will be included in future studies because of the increasing of already resistant isolates from clinical specimens (Bongiorno et al., 2018). However, further studies, particularly using *in vivo* models, are required to confirm its effectiveness.

## Data Availability Statement

The raw data supporting the conclusions of this article will be made available by the authors, without undue reservation, to any qualified researcher.

## Author Contributions

AG, SS, DF, FB, and CM contributed conception and design of the study. AG and SS performed the experiments and wrote the first draft of the manuscript. AG, SS, SP, and ES contributed acquisition, analysis, and interpretation of the data. AG, SS, FB, and CM wrote sections of the manuscript. All authors contributed to the critically manuscript revision, read, and approved the submitted version.

## Conflict of Interest

The authors declare that the research was conducted in the absence of any commercial or financial relationships that could be construed as a potential conflict of interest.
